# Preliminary validation for an online configuration determination method of a thin film buckling under point contact force

**DOI:** 10.1038/s41598-024-83849-8

**Published:** 2024-12-30

**Authors:** Yeoun-Jae Kim, Daehan Wi

**Affiliations:** Biomedical Engineering Research Center, Asan Institute for Life Sciences, Asan Medical Center, 88, Olympic-ro 43-gil, Songpa-gu, Seoul, 05505 South Korea

**Keywords:** Thin film, Point contact force, Buckling configuration, Statics, Constrained optimization, Ansys simulation, Experiment, Biomedical engineering, Mechanical engineering

## Abstract

The authors previously developed an online thin film buckling configuration determination method for a mini basket type mapping catheter prototype, which incorporates eight thin film sensor strips. In the prior study, no external force was applied to the thin film, and only axial displacement was adjusted to induce buckling in the thin film. Extending this prior work, a preliminary methodological validation is conducted for an online configuration determination method of thin film buckling under a point contact force. The overall thin film configuration determination problem is formulated as a constrained optimization problem, involving five variables and five equality constraint functions. Before developing an actual online optimization solver, preliminary numerical calculations, Ansys simulations, and experiments are performed to verify the proposed problem formulation. The comparison between the numerical precalculations, Ansys simulations, and experimental results demonstrated that the proposed problem formulation is consistent with Ansys simulations and experimental outcomes. This indicates that the proposed formulation is capable of calculating accurate solutions using appropriate optimization methodologies.

## Introduction

Thin films are used in various application areas such as coating^1,2^, flexible sensors / biosensors with integrated passive devices and circuits^3,4^,^5^, thin film photovoltaic cells^6^, and thin film batteries^7^. For biomedical application, various thin films are developed and manufactured with different chemacal composition and coating technology^8–11^. In medical thin film flexible sensor application, mini basket type mapping catheters^12–14^ are used for arrhythmia intervention procedure to localize the arrhydimia source by measuring electrocardiography signal on heart. It consists of multiple printed circuit board (PCB) integrated thin films, which can be adjusted to be flattened to be introduced inside of heart through femoral vein. After it has been introduced to the heart, it is buckled, translated and rotated to make the electrode tips on the thin films be contact with the cardiac surface and measure electrocardiography (ECG) signal.

**Figure 1:**
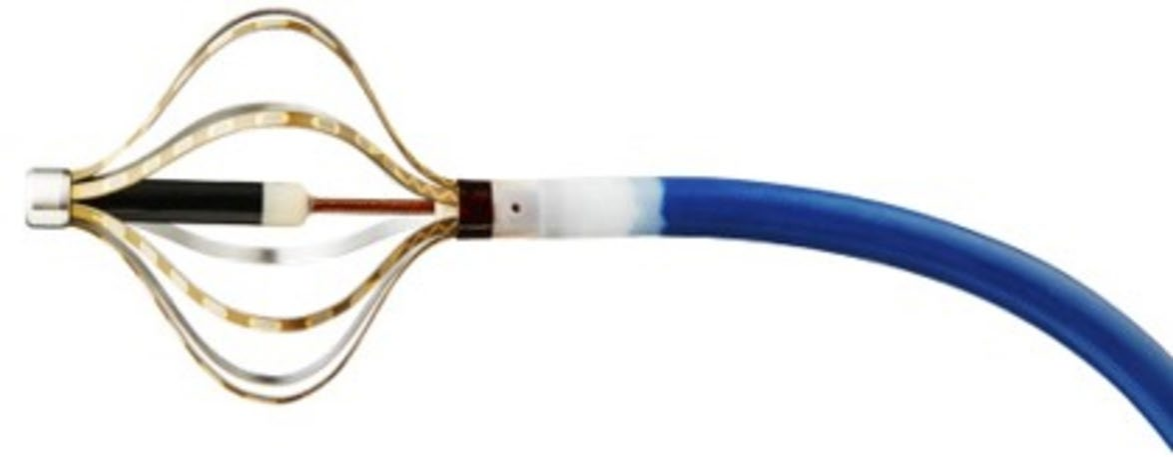
Developed mini basket type mapping catheter prototype.

The authors and colleagues previously made a mini basket type mapping catheter prototype to address the possibility of clinical usage as depicted in Fig. 1. The mapping catheter prototype consists of eight thin film PCB sensor strips, which has eight electrodes to measure the ECG signal on the surface of the heart. The two boundaries of the thin film PCB sensor strip in Fig. 1 can be adjusted to be buckled, translated and rotated to be in contact with the cardiac surface as a commercial mini basket type mapping catheter does. However, to accurately localize the ECG signal source with the mapping catheter prototype, an accurate buckled thin film configuration must be clarified beforehand. Therefore, in the previous work^15^, an online thin film buckling configuration determination method was proposed for flexible sensor application by authors. With the proposed method, the precise positions of the electrodes on the thin film in Fig. 1 with respect to the base coordinates of the mapping catheter prototype can be determined. However, when the deflected film sensor has contact with the surface wall on the heart, the film sensor is deflected according to the applied force on the sensor. The previously proposed method was developed with no contact force on the thin film configuration. Therefore, to more accurately localize the sensor strip configuration with external force, a online configuration determination of a thin film buckling under point contact force must be developed.

In this work, an online configuration determination of a thin film buckling under point contact force is formulated as a constrained optimization problem, which has five variables and five equality constraints to minimize. However, before developing the online solver for the optimization problem, preliminary validation of the problem formulation must be conducted beforehand to minimize the problem formulation errors and reduce the validation burden of future online solver development. Therefore, preliminary numerical calculation for the solution of problem formulation, Ansys simulation^16^, and experiment are performed to verify the proposed problem formulation by determining approximate solutions.

### Relates works

The thin film buckling under a point contact force can be categorized as buckling of a slender superelastic material with various boundary and force conditions. Traditionally, they are solved by exact solution using Euler’s elastica^17–25^ or approximate solution from perturbation theory^26,27^. Timoshenko et al.^17^ induced Elastica based solution of column buckling problem with no lateral forces in clamped boundary. Hubbard^18^ also developed the solution of pole-vaulting problem, in which lateral force exists with axial force in clamped boundary condition. Griner^19^ also solved the pole-vaulting problem by parametric solution with tabulated elliptic integral. Mikata^20^ introduced an exact solution of clamped-hinged beam buckling problem with Elastica for carbon nanotube application.

Armanini et al.^21^ determined an Elastica based solution for superelastic compass and catapult under the boundary condition of a point force at one end and rotation at the other end. Plaut et al.^22^ make the straight elastica bent in clamped boundary condition until its ends are at vertical and pushed the bent elastica to the ground to investigate the configuration. Chen et al.^23^ investigated a buckled elastica under off-axis point constraint in clamped boundary condition. They compared the numarical calculation with experimental result.^24^ also induced buckled configuration under point contact force in pinned boundary condition and validated their solution with experiment. In^25^, Chen et al. induced deformation of pressed elastica in clamped boundary condition with experimental results.

Compared with Euler’s Elastica based method, which integrates the buckling/deflection configuration along axial body coordinates, an approximate solution by purturbation theory incorporates a parametric purturbative expansion and determines each parameters by iteratively inserting the expansion into the original govering equation. Berkey et al.^26^ applied the purturbation method to solve the buckling of compressed elastic column with pinned boundary condition. Wang^27^ proposed the solution of an inclined cantilaver with an end load by using purturbation method.

Finite element method (FEM) can also be used to solve the buckling problems. There are many commercial^16,28–30^ and open source software available^31–33^. However, using these software to solve the buckling problem can be expensive for commercial software and need additional validation for open source software, which make the FEM method not suitable for online buckling configuration determination.

### Contributions

In force and boundary condition perspective, this work determines elastica configuration under point contact force with various force applying angle in clamped boundary condition for a mini basket type mapping prototype application. Its boundary and force condition differ from references^17–25^ or approximate solution from perturbation theory^26,27^. In these references, Chen et al.^23–25^solved similar elastica problems, however, with a fixed point constraint under clamped boundary condition^23^, vertical point contact force under pinnded boundary condition^24^, and vertically pressed force condition in clamped boundary condition^25^. To the best of knowledge, there has been no works in which thin film buckling problem is formulated to single objective optimization problem for online configuration calculation. With the proposed optimization framework, the thin film configuration under point contact force can be solved by utilizing the conventional iterative constrained optimization methods, such as penalty function method, Lagrange multiplier method^34^, sequential quadratic programming (SQP)^35^, and hybrid projection method^36^ without directly designing and implementing the solver. Moreover, incorporating the regularization method in the optimization framework, such as Tichonov regularization^36^, can cope with the measurement uncertainty in the deflected configuration.

The main contributions of this paper are summarized below.

1. An online configuration determination method of a thin film buckling in clamped boundary under a point contact force is formulated as constrained optimization problem by connecting four buckled configurations.
2. Preliminary numerical calculation for the solution of the proposed problem formulation is conducted and compared with Ansys simulations and experimental results with screen protector to address the validity of the proposed problem formulation by determining an approximate solution of the problem.

The problem definition and constrained optimization problem formulation are explained in section 2. In section 3, preliminary numercal calculation, Ansys simulation, experimental result with screen protector are discussed and compared to verify the problem formulation. Discussion and conclusion is set forth in section 4 with future works.

## Thin film buckling configuration determination method

### Preliminaries

**Figure 2:**
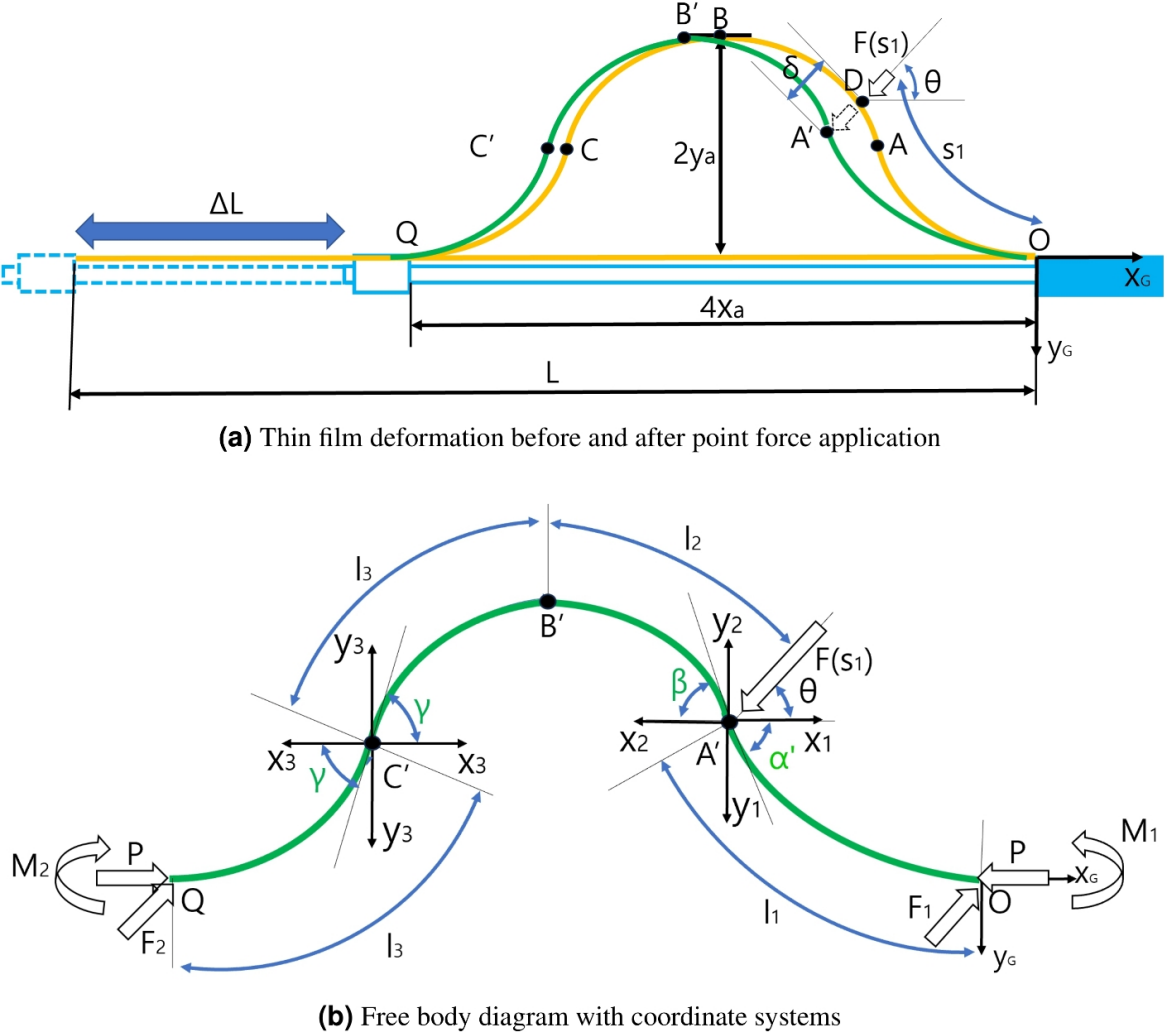
Schematics of thin film buckling under point contact force.

The schematics and free body diagram of thin film buckling in the mapping catheter prototype in Fig. 1 are presented in Fig. 2a, b, respectively.

A quarter model of the thin film strip sensor in Fig. 2a determines the overall configuration because the overall configuration is y axis symmetry at $2\textit{x}_{a}$ position and point symmetry at $\textit{x}_{a}$ position.

## Problem formulation

### Overall and segmentwise configuration analysis

The schematics of thin film buckling under point contact force is presented in Fig. 2a, in which the mapping catheter moving rod is represented in sky blue, which is assembled to the main body in the rightmost side and fixed horizontally. A thin film strip in Fig. 2a is represented in yellow, which is fixed at both ends of the moving body and its total length is *L*. If the length of the moving rod is shortened to $\Delta$*L* as depicted in Fig. 2a, the configuration of thin film strip changes from straight yellow line to yellow buckled shape, with $4\textit{x}_{a}$ width and $2\textit{y}_{a}$ height, respectively. The coordinate systems in Fig. 2a, attached at point O, represents the global coordinates (O$_{G}$-x$_{G}$-y$_{G}$), which represents the base coordinates of the whole configuration of the thin film strip sensor. Q is the end point of the buckled configuration. As can be seen in Fig. 1, the buckled configuration is symmetric at its midpoint and axisymmetric at its quarter and third quarter point. Therefore, the yellow buckled configuration in Fig. 2a is symmetric at Point B and axisymmetric at Point A and C, which are midpoint, A quarter, and third quarter point of thin film length, respectively. As a result, the yellow buckled configuration can be divided into four identical segments.

In Fig. 2a, with the yellow deformed configuration, the force $F(s_1)$ is applied with $\theta$ inclination angle on Point D, at which the thin film length is $s_1$ from point O. After the force application, the yellow buckled thin film changes its configuration to green curve as depicted in Fig. 2a. During the force $F(s_{1})$ application, the force inclination angle $\theta$ is fixed. However, the force application point can be changed by slipping of the force contact point on the thin film, which is point A$'$ in Fig. 2a on the green curve. The distance between point A$'$ and D is presented as $\delta$ as depicted Fig. 2a.

Note that After static equilibrium is reached as a result of the force application, the green O-A$'$-B$'$-C$'$-Q configuration under point contact force is not symmetric, contrary to the doubly symmetric yellow O–A–B–C–Q configuration. The initial and force applying condition and the resultant configuration assumptions of thin film buckling under point contact force are summarized below.

1. $\Delta$ *L* in Fig. 2a is adjusted for the deflection angle at Point A is 45$^\circ$, in which the applied axial force in x$_{G}$ direction in Fig. 2a is *P*.
2. F is applied at $s_1$ position (D in Fig. 2a) in thin film body coordinates with force applying angle $\theta$ maintained. The force application position is between Point A and B in Fig. 2a (0.25*L* < $s_1$ < 0.5*L*). The force application point $s_1$ position (D in Fig. 2a) in thin film body coordinates can be changed by slipping of the force contact point on the thin film.
3. At Point A$'$, which is the final force application point in Fig. 2a, static equailbrium is reached. The distance between Point A$'$ and D is $\delta$.
4. The final green configuration in Fig. 2a can be divided into OA$'$, A$'$B$'$, B$'$C$'$, and C$'$Q segments. Contrary to initial yellow configuration in Fig. 2a, these segments are not identical except B$'$C$'$, and C$'$Q segments, which are axisymmetric at point C$'$.
5. A$'$ point of the final green configuration in Fig. 2a is piecewise continuous inflection point. Also, B$'$ point is continuous and C$'$ point is continuous inflection point.

The free body diagram with coordinate systems for analyzing the green configuration is presented in Fig. 2b. x$_1$-y$_1$, x$_2$-y$_2$, x$_3$-y$_3$, and x$_4$-y$_4$ coordinates are attached at A$'$, C$'$ points in Fig. 2b for analyzing each segments OA$'$, A$'$B$'$, B$'$C$'$, and C$'$Q, which are segment coordinates. The buckling angle of each four segments with respect to each segment coordinates are $\alpha$ $'$ for A$'$B$'$ segment, $\beta$ for B$'$C$'$ segment, and $\gamma$ for B$'$C$'$, and C$'$Q segment, as depicted in Fig. 2b. $l_1$, $l_2$, $l_3$, and $l_4$ in Fig. 2b are the length of each segment. The force *P* at point O and Q in the free body diagram in Fig. 2b is the axial force to make the initial buckled configuration and $F_1$, $M_1$, $F_2$, $M_2$ are reactive forces and moments at point O and Q by the external force $F(s_1)$ by static equilibrium in overall O–A$'$-B$'$-C$'$-Q configuration.

**Figure 3:**
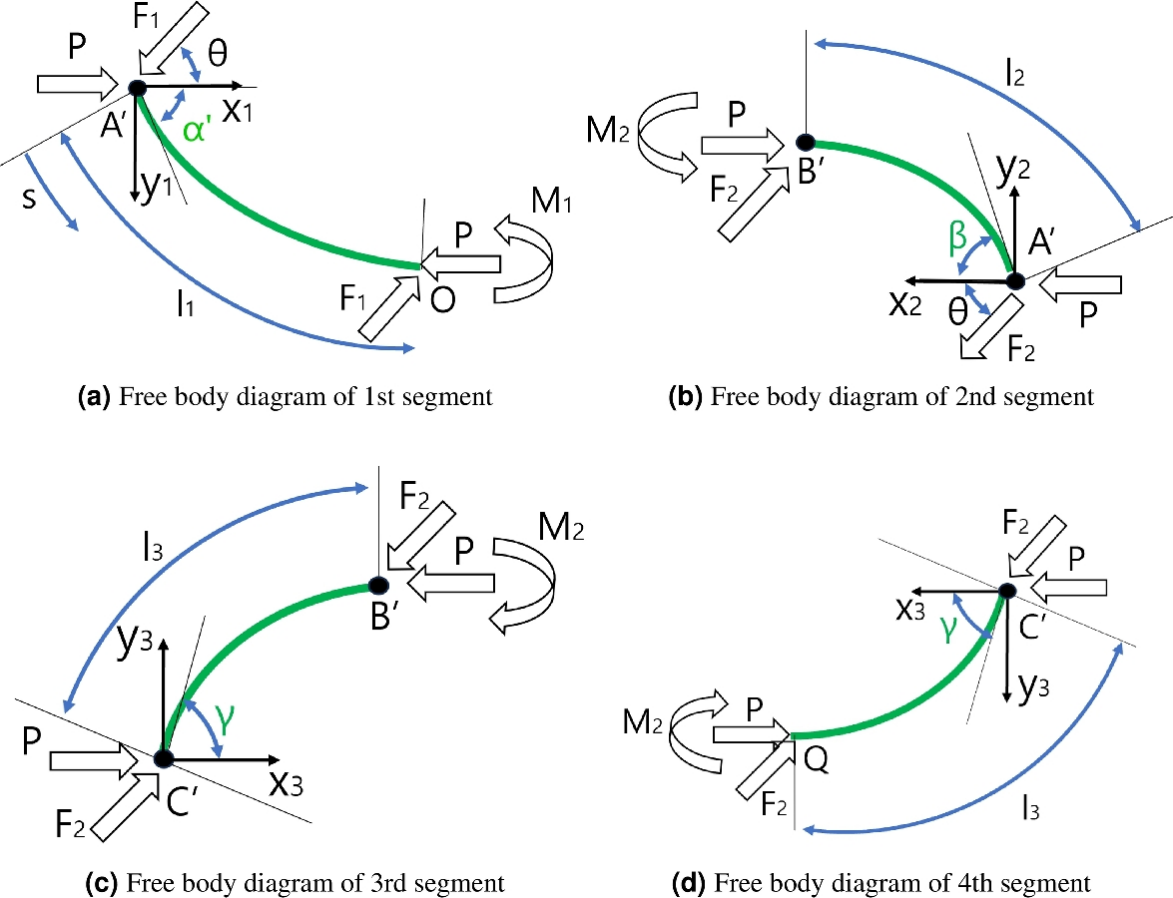
Free body diagrams of four segments of the buckled thin film under point contact force.

The free body diagram in Fig. 2b can be divided by segmentwise freebody diagrams, which presents the free body diagrams of 1st segment - 4th segment, as depicted in Fig. 3a–d. Because the overall buckled thin film is in static equilibrium, the each four segments must also be in static equilibrium and their free body diagram can be drawn and analyzed. The external force $F(s_1)$ can be divided to $F_1$ and $F_2$, which presents the external force applied at 1st segment and 2nd segment, respectively. In the free body diagram of 1st segment in Fig. 1a, $F_1$ and *P* are applied at A$'$ point and $F_1$, *P*, and $M_1$ are applied at point O by force and moment equilibrium, as described in Fig. 3a. By the observation 2, the moment at A$'$ point is zero and the moment by *P* and $F_1$ with respect to point O equals $M_1$ in Fig. 3a.

The free body diagram of 2nd segment in Fig. 3b can also be constructed by continuity of force / moment at point A$'$ and force / moment equilibrium. There are compressive force *P* at point A$'$ and B$'$ and force $F_2$ is applied to point A$'$ because it is the external force applied at 2nd segment. Because the moment at point A$'$ is zero, the bending moment $M_2$ must be applied at point B$'$. The free body diagram of 3rd and 4th segments in Fig. 3c, d can be constructed in a similar manner.

### Segmentwise and overall configuration determination

The solution of initial configuration before force application, which is yellow curve in Fig. 2a can be determined by Euler’s Ealstica based method^15^^17^. By authors in the previous work^15^, the configuration of A quarter model, which is yellow OA curve in Fig. 2a, is solved by integrating the large deflected finite element based on external force *P* and initial deflection angle $\alpha$, which are the independent variables for the configuration determination. With the configuration of the quarter model, whole configuration is constructed by copying the quater model axisymmetrically and symmetrically. However, after applying the external force $P(s_1)$, each four segments in Fig. 3a–d must be solved individually utilizing the deflection equation because the configuration is not symmetric. Also, there are lateral forces $F_1$ and $F_2$ applied at each segmemt alongside the axial force *P* and initial bending angle $\alpha$, which is not take account in the pervious work^15^.

Hubbard^18^ solved this problem for the pole vaulting application. With axial force *P*, lateral force *F*, and initial bending angle $\alpha$ in the vaulting pole, the configuration of the vaulting pole is calculated by integrating the deflection equation along the deflected configuration. In this work, this solution procedure is adopted for the configuration determination of each segment in Fig. 3a–d with slight modification of the original solution. The solution procedure for the configuration calculation of each segment is as follows.

1. Reformulate the problem in Fig. 3a–d, in which the segment under the axial force *P*, lateral force $F_1$($F_2$), and initial bending angle $\alpha '$ ($\beta$ and $\gamma$) to the segment under the axial force *Q* and initial bending angle $\eta$.
2. Solve reformulated problem with axial force *Q* and initial bending angle $\eta$ by integrating the deflection equation along the configuration as described in the researches^15,17^.

In Fig. 3a, for the reformulation of the problem, which determines the configuration of segment A$'$O under axial force *P*, lateral force $F_1$, and initial bending angle $\alpha '$, the forces are decomposed to the x$_1$ directional axial force ${{\hat{P}}}$ and y$_1$ directional lateral force ${\hat{F}}$ as presented in Eqs. (1) and (2).

1 $$\begin{aligned} {\hat{P}}= & P - {F_1}\cos \theta \end{aligned}$$

2 $$\begin{aligned} {\hat{F}}= & {F_1}\sin \theta \end{aligned}$$

For the initial bending angle change, the angle between ${\hat{F}}$ and ${\hat{P}}$ is calculated in Eq. (3) and summed with the iniital bending angle $\alpha '$ to calculate the changed initial bending angle $\eta$, which is described in Eq. (4).

3 $$\begin{aligned} & \psi = {\tan ^{ - 1}}( - \frac{{{\hat{F}}}}{{{\hat{P}}}}) \end{aligned}$$

4 $$\begin{aligned} & \alpha ' + \psi = \eta \mathrm{ } \end{aligned}$$

The overall applied axial force *Q* can be calculated by Eq. (5).

5 $$\begin{aligned} \sqrt{{{{\hat{P}}}^2} + {{{\hat{F}}}^2}} = Q\mathrm{ } \end{aligned}$$

With *Q* and $\eta$, the configuration of segment can be determined as those with only axial force *P* and initial deflection angle $\alpha$, which is described in the researches^15,17^. For the integration of the deflection equation along *s* in Fig. 3 (a), the axial force *Q* and the initial bending angle $\eta$ is changed to *k* and *p* variables by Eqs. (6) and (7).

6 $$\begin{aligned} k= & \sqrt{\frac{Q}{{EI}}} \end{aligned}$$

7 $$\begin{aligned} p= & \sin (\frac{\eta }{2}) \end{aligned}$$

In Eq. (6), *EI* is the flexural rigidity of the thin film. ${\hat{\theta }}$ in Eq. (8) is the tangent angle of configuration (a) with respect to x$_1$ axis at s position, which is used as integration variable. $\phi$ in Eq. (8) is changed variable made out of ${\hat{\theta }}$ to change the limit of integral. $\phi ^*$ in Eq. (9) is the upper limit of integral.

*k* and *p* in Eqs. (6) and (7) are the changed variables with *Q* and $\eta$. As described in the work^17^, the integration along the body coordinate s in Fig. 3a is performed to decide the segment configuration. ${\hat{\theta }}$ in Eq. (8) is the tangent angle of segment configuration in Fig. 3a with respect to x$_1$ axis at s position, which is used as integration variable. $\phi$ in Eq. (8) is changed variable made out of ${\hat{\theta }}$. $\phi ^*$ in Eq. (9) is the upper limit of integral.

8 $$\begin{aligned} \sin \frac{{{\hat{\theta }} }}{2}= & p\sin \phi = \sin (\frac{\eta }{2})\sin \phi \end{aligned}$$

9 $$\begin{aligned} {\phi ^*}= & {\sin ^{ - 1}}(\frac{\psi }{{2p}}) \end{aligned}$$

If the integration is performed, (x, y) position and length s at $\phi ({\hat{\theta }} )$ of the green configuration in Fig 3a can be calculated, as described in Eqs. (10) and (11). $l_1$ in Eq. (12) is the overall length in Fig. 3a. *E*(*p*), *F*(*p*), $E(\phi |p)$, and $F(\phi |p)$ in Eqs. (10) and (11) are complete elliptic integral of 2nd and 1st kind and incomplete elliptic integral of 2nd and 1st kind, respectively.

10 $$\begin{aligned} & \begin{array}{l} \left[ {\begin{array}{*{20}{c}} x\\ y \end{array}} \right] = \left[ {\begin{array}{*{20}{c}} \begin{array}{l} \displaystyle {((\frac{2}{k}E(p) - \frac{1}{k}F(p) - (\frac{2}{k}E(\phi ({\hat{\theta }} )|{p^2})} \displaystyle {- \frac{1}{k}F(\phi ({\hat{\theta }} )|{p^2})))\cos \psi + \frac{{2p}}{k}\cos \phi ({\hat{\theta }} )\sin \psi )} \end{array}\\ \\ \begin{array}{l} \displaystyle {( - (\frac{2}{k}E(p) - \frac{1}{k}F(p) - (\frac{2}{k}E(\phi ({\hat{\theta }} )|{p^2})} \displaystyle {- \frac{1}{k}F(\phi ({\hat{\theta }} )|{p^2})))\sin \psi + \frac{{2p}}{k}\cos \phi ({\hat{\theta }} )\cos \psi )} \end{array} \end{array}} \right] \end{array} \end{aligned}$$

11 $$\begin{aligned} & s(\phi ({\hat{\theta }} )) = \frac{1}{k}K(p) - \frac{1}{k}F(\phi ({\hat{\theta }} )|{p^2}) \end{aligned}$$

12 $$\begin{aligned} & {{l_1} = s({\phi ^*}) = \frac{1}{k}K(p) - \frac{1}{k}F({\phi ^*}|{p^2})} \end{aligned}$$

The other configurations in Fig. 3b–d can be calculated with Eqs. (10)–(12) accordingly with changed *k*, *p*, and $\phi ^*$ values. The overall green configuration of Fig. 2b can be constructed by attaching the 4 calculated segment configurations at A$'$, B$'$, and C$'$.

### Problem formulation with overall configuration calculation

In the previous subchapter, the configuration of each segments in Fig. 3a–d and overall configuration in Fig. 2b can be determined, in case the thin film is deflected by moving rod movement ($\Delta L$) and external force $F(s_1)$ in Fig. 2a. However, there are many unknown for the configuration determination, which is summarized below with known.

1. Known: $\Delta L$, $F(s_1)$($F_x$, $F_y$), $\theta$, and the initial configuration without external force including *P*, $x_a$ and $y_a$ in Fig. 2a, b.
2. Unknown: initial bending angles ($\alpha '$, $\beta$, $\gamma$), the lateral forces ($F_1(F_{1x}, F_{1y})$, $F_2(F_{2x}, F_{2y})$), and moments ($M_1$, and $M_2$) in static equilibrium under external force $F(s_1)$ in Fig. 3a–d

Because there are nine unknown, nine equality constraints must be constructed to determine the unknowns. By force equilibrium of the overall configuration in Fig. 2b and moment equilibrium at point A$'$ and C$'$ in Fig. 3a, d, respectively, the following Eqs. (13)–(16) can be made.

13 $$\begin{aligned} & {F_{1x}} + {F_{2x}} = F\cos \theta = {F_x} \end{aligned}$$

14 $$\begin{aligned} & {F_{1y}} + {F_{2y}} = F\sin \theta = {F_y} \end{aligned}$$

15 $$\begin{aligned} & {M_1} + ({F_{1x}} - P){y_{1a}} - {F_{1y}}{x_{1a}} = 0 \end{aligned}$$

16 $$\begin{aligned} & {M_2} - ({F_{2x}} + P){y_{4a}} + {F_{2y}}{x_{4a}} = 0 \end{aligned}$$

By using above equations, $F_2(F_{2x}, F_{2y})$, $M_1$, and $M_2$ can be calculated with $F_{1x}$ and $F_{1y}$. Therefore, there are five unknown $\alpha '$, $\beta$, $\gamma$, $F_{1x}$, $F_{1y}$. Moreover, the overall thin film length *L* in Fig. 2a must equal the summation of the length of the segments in Fig. 3a–d, as described in Eq. (17). The overall x directional and y directional displacements of the deflected film (4$x_a$ and 2$y_a$) in Fig. 2a must equal the summation of the x directional and y directional displacements of 4 segments in Fig. 3a–d, which are in Eqs. (18) and (19).

17 $$\begin{aligned} & \sum \limits _{n = 1}^4 {{l_n} = L} \end{aligned}$$

18 $$\begin{aligned} & {y_{1a}} + {y_{2a}} = {y_{3a}} + {y_{4a}} = 2{y_{a}} \end{aligned}$$

19 $$\begin{aligned} & \sum \limits _{n = 1}^4 {{x_{na}} = 4{x_a}} \end{aligned}$$

By the continuity at point B$'$ in Fig. 2b, the momemnt at point B$'$ in 2nd and 3rd segments must be equal. This constraint is in Eq. (20).

20 $$\begin{aligned} {\left. {{M_2}} \right| _{\mathrm{{@segment 2}}}} = {\left. {{M_2}} \right| _{\mathrm{{@segment 3}}}} \end{aligned}$$

The last constraint is the work-energy constraint. The work applied to the thin film by external force $F(s_1)$ must equal the energy stored in the deflected configuration, which is expressed in Eq. (21).

21 $$\begin{aligned} \displaystyle {\frac{{F\delta }}{2} = \sum \limits _{n = 1}^4 {\int \limits _{s = 0}^{{l_n}(s)} {\frac{{{M^2}(s)}}{{2EI}}} } ds - {E_i}} \end{aligned}$$

$E_i$ in Eq. (21) is the total bending energy in the yellow configuration before force application and $\displaystyle {\frac{{{M^2}(s)}}{{2EI}} ds}$ is bending moment energy in *ds* element in each segment, which is integrated along each segment’s length $l_n(s)$. By subtrcting the summation of the bending moment energy in each segment from $E_i$, the stored energy can be calculated as described in the right hand side of Eq. (21). $\displaystyle {\frac{F \delta }{2}}$ in the left side of Eq. (21) is the work applied by the external force, in which $\delta$ is the displacement between point D and A$'$ in Fig. 2a, which can be calculated with the green final configuration in Fig. 2a.

With five variables ($\alpha '$, $\beta$, $\gamma$, $F_{1x}$, $F_{1y}$) and five constraints (Eqs. (17)–(21)), there could be many solutions to satisfy all the constraints. In this study, the solution, which minimizes the external force applying distance $\delta$ is defined as the optimal solution for the configuration determination. The problem formulation is in Eq. (22).

22 $$\begin{aligned} \begin{array}{l} \mathrm{{minimize: }}\hspace{5 mm}\delta \\ \mathrm{{over: }}\hspace{5 mm}\alpha ',\hspace{1 mm}\beta ,\hspace{1 mm} \gamma ,\hspace{1 mm} {F_{1x}},\hspace{1 mm} {F_{1y}}\\ \mathrm{{subject \hspace{1 mm} to: }}\\ \hspace{5 mm}\mathrm{ }\sum \limits _{n = 1}^4 {{l_n} - L = 0} \\ \\ \hspace{5 mm}\mathrm{ }{y_{1a}} + {y_{2a}} - 2{y_{3a}} = 0\\ \hspace{5 mm}\mathrm{ }\sum \limits _{n = 1}^4 {{x_{na}} - {x_a} = } 0\\ \\ \hspace{5 mm}{\left. {\mathrm{ }{M_2}} \right| _{\mathrm{{@segment 2}}}} - {\left. {{M_2}} \right| _{\mathrm{{@segment 3}}}} = 0\\ \\ \hspace{5 mm}\mathrm{ }\displaystyle {\frac{{F\delta }}{2}} - (\sum \limits _{n = 1}^4 {\int \limits _{s = 0}^{{l_n}(s)} {\frac{{{M^2}(s)}}{{2EI}}} } ds - {E_i}) = 0 \end{array} \end{aligned}$$

## Experimental result, preliminary numerical calculation, and Ansys simulation of screen protector under external force

### Experimental result

The resultant configuration assumptions of the thin film buckling under point contact force, which are summarized in 4. and 5., states that all the segment’s configuration in Fig. 3a–c must be purely convex, without any inflection points or linear subsegments. To validate this convexity assumption, 27 experiments using a screen protector are performed with respect to $s_1$ (force applying position), $\theta$ (force applying angle), and $F(s_1)$ (force magnitude) combinations in Fig. 2a. the force applying position $s_1$ are set to 5, 10, and 15 mm from point A$'$ in Fig. 2b, which are a 1/3, 2/3, and 3/3 of the 2nd segment’s length, which meent the force applying condition ($0.25L< s_1 < 0.5L$). Also, the force applying angle $\theta$ are 10, 45, 80$^\circ$ and and the force magnitude $F(s_1)$ are set to 0.83, 1.66, and 2.5 g, respectively, which are 1/3, 2/3, and 3/3 of initial axial force *P* in Fig. 2b.

**Figure 4:**
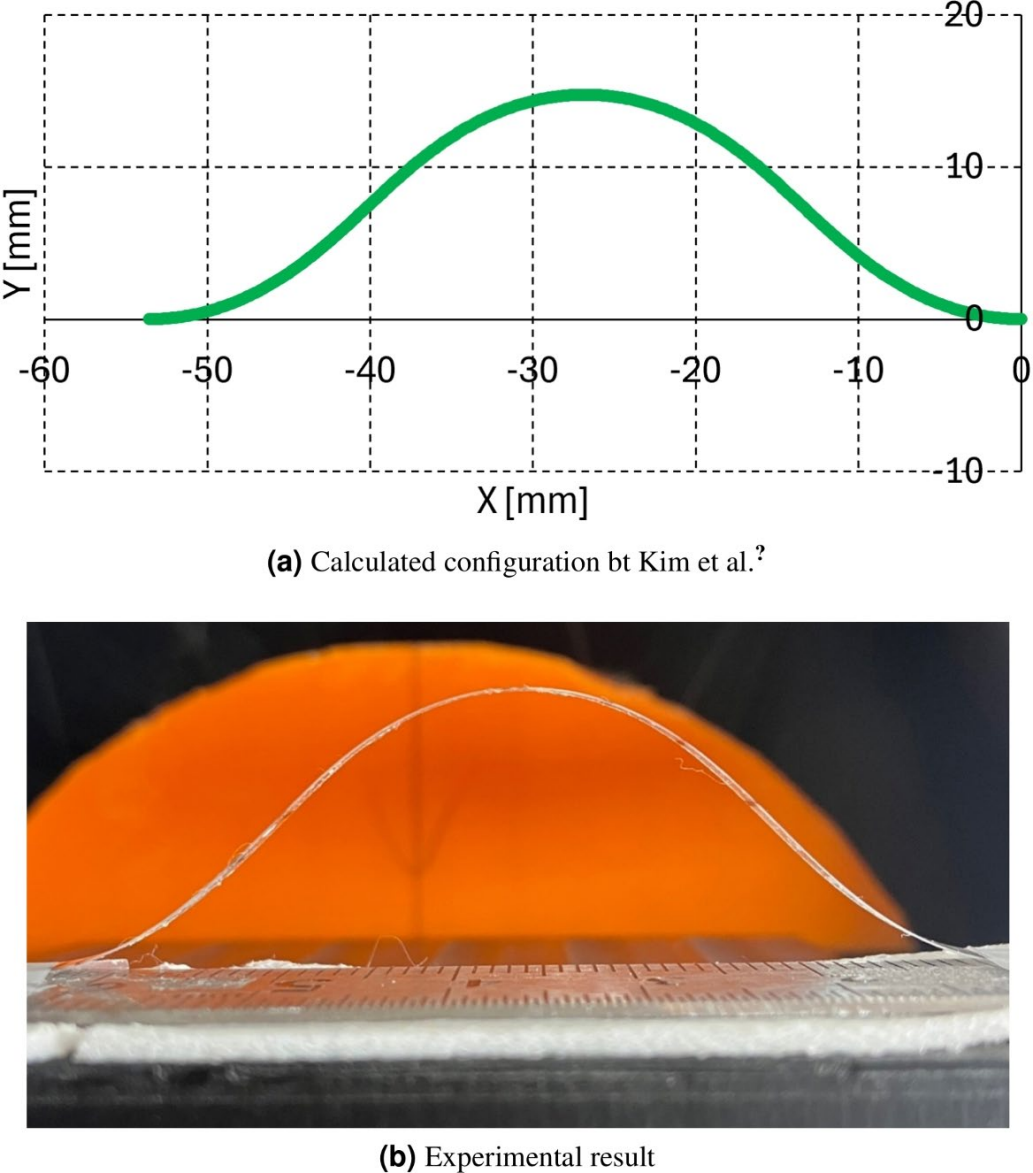
Initial configuration without external force.

The dimensions of the screen protector (polyethylene material) are as follows: thickness = 0.11 mm, width = 4.0 mm, and length = 62.95 mm, consistent with those reported by Kim et al.^15^. The modulus of elasticity of the screen protector is 5 GPa, according to local vendor test results. Using the method proposed by Kim et al.^15^, the initial buckling configuration without external force is calculated, which results in 4$x_a$ = -53.24 mm, 2$y_a$ = -14.73 mm, and *P* = 2.5 g with $\alpha$ $'$, $\beta$, $\gamma$ = 45$^\circ$ in Fig. 2a, b, which is the initial condition 1. in section 1.1. The screen protector is buckled with 4$x_a$ = 53.2 mm according to the calculation result and the measured $\alpha$ $'$ and 2$y_a$ values are approximately 45$^\circ$ and 16.0 mm, which are consistent with the calculation results with 1.3 mm 2$y_a$ value error between the calculation and experiment. The calculated and experimental results are presented in Fig. 4a, b.

**Figure 5:**
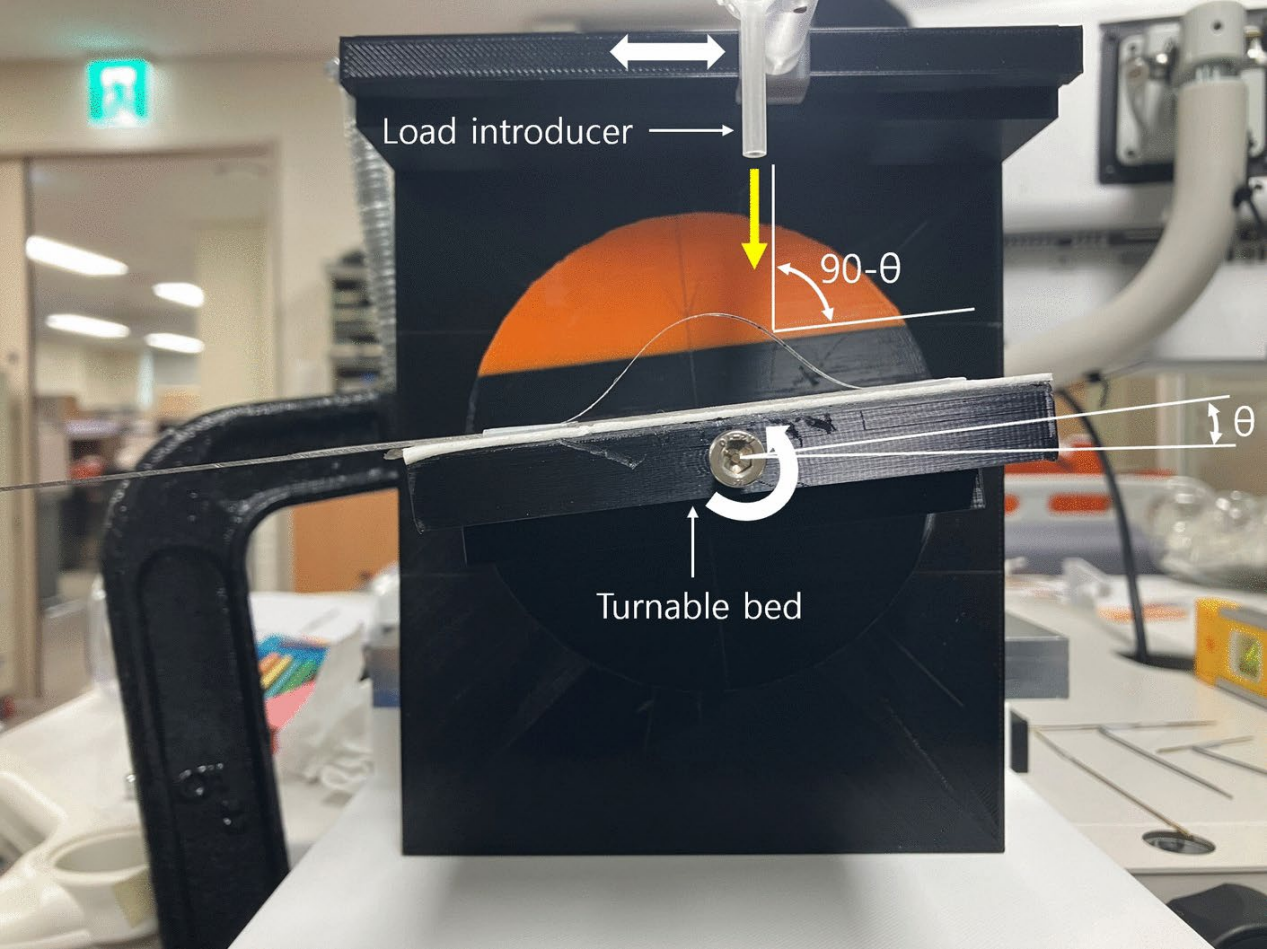
Experimental setup.

The experimental setup is depicted in Fig. 5. The initially buckled screen protector in Fig. 4b is placed on a turntable bed in Fig. 5. By rotating the turntable bed counterclockwise at $\theta$ $^\circ$, as indicated by the white curved arrow in Fig. 5, the force applying angle can be set to 90$^\circ$-$\theta$ $^\circ$ as depicted in Fig. 2a. The force applying position $s_1$ can be adjusted by horizontally moving the load introducer in Fig. 5 as indicated by white arrow. Slim rods with 0.83 g, 1.66 g, and 2.5 g weigt are introduced to the load introducer to apply the fore $F(s_1)$ to the buckled screen protector. The load direction is indicated by yellow arrow in Fig. 5.

The results are summarized in Table 1. In Table 1, the $\theta$, $s_1$, and $F(s_1)$ columns present the experimental conditions, and the $\alpha$ $'$, $\beta$, and $\gamma$ columns present the measured $\alpha$ $'$, $\beta$, and $\gamma$ values under each experimental condition, as determined by graphical analysis. The configuration column represents the configuration characteristics of each segment, which are convex, s-shape, slight s-shape, and N/A. Convex means that all the configuration of four segments are purely convex, as depicted in Fig. 3a–d. S-shape means that one of the four segments is s-shape, which has an inflexion point. A slight s-shape configuration is that one of the four segments is slight s-shape, which has an inflexon point close to the end point of the segment. N/A means that the configuration can not be determined because the experiment is infeasible by slipping of the force applying rod on the thin film or the deformation is too little to determine the deformed configuration. Note that $F(s_1)$ values are slightly different in each condition because of the change of the force applying slim rod and the $\alpha$ $'$, $\beta$, and $\gamma$ values are obtained only when the configuration is convex or slightly s-shape.

**Table 1:** Experimental result summary.

$\theta$	*s*1	$F(s_1)$	$\alpha '$	$\beta$	$\gamma$	Configuration
$10^{\circ }$	5	0.85	37.5	35	44	Convex
		1.55	38.3	34	49	Convex
		2.42	39	39	54	Convex
	10	0.85	–	–	–	N/A
		1.55	26	21	51	Slight s-shape
		2.42	27	26	53	Slight s-shape
	15	0.85	–	–	–	N/A
		1.55	–	–	–	N/A
		2.42	–	–	–	s-shape
$45^{\circ }$	5	0.85	41	40	48	Convex
		1.55	41	41	48.5	Convex
		2.42	38	40.5	50.5	Convex
	10	0.85	–	–	–	s-shape
		1.55	37	38	55.5	Convex
		2.42	35	35	53	Convex
	15	0.85	–	–	–	N/A
		1.55	–	–	–	N/A
		2.42	–	–	–	s-shape
$80^{\circ }$	5	0.85	43.5	40	44	Convex
		1.61	39	40	50	Convex
		2.52	30	43	48	Slight s-shape
	10	0.85	–	–	–	s-shape
		1.61	42	40	50	Convex
		2.52	40	49	50	Convex
	15	0.85	–	–	–	N/A
		1.55	–	–	–	N/A
		2.42	–	–		N/A

$\alpha$ $'$, $\beta$, and $\gamma$ values in Table 1 are quite diverse. At $s_1$ = 15.7 + 15 mm, all the configurations are N/A or s-shape. At $s_1$ = 15.7 + 10 mm, the configurations vary, including N/A, slight-s shape, s-shape and convex. When $s_1$ = 15.7 + 5 mm, all the configurations are convex except $\theta$ = 80$^\circ$, $F(s_1)$ = 2.52 g, in which the configuration is slightly s-shape. According to Table 1, the problem formulation in Eq. (22) is feasible only when $s_1$ $\le$ 15.7 + 5 mm and $F(s_1)$ $\le$ *P*, and $\theta$ $\le$ 45$^\circ$.

**Figure 6:**
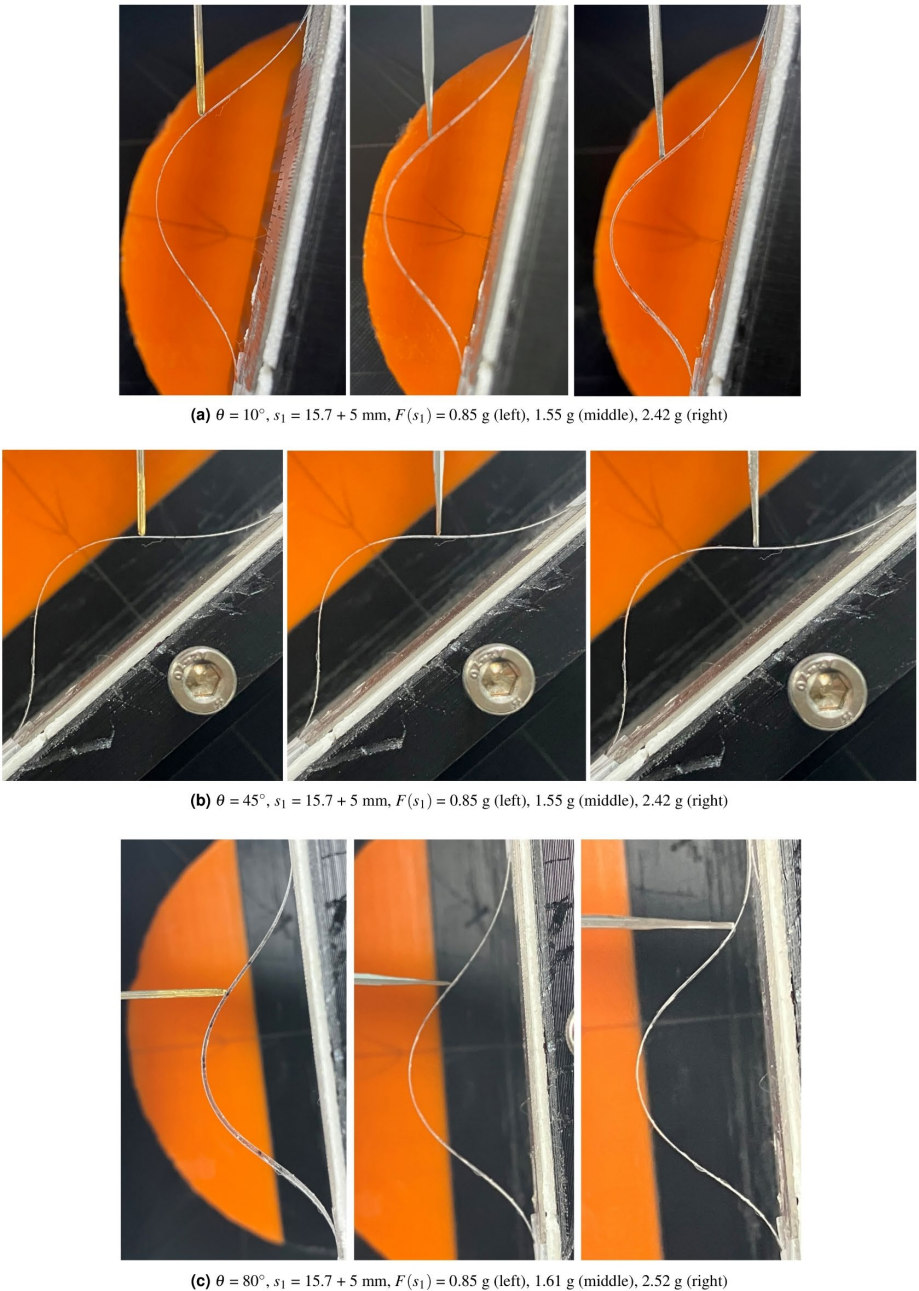
$s_1$ = 15.7 + 5 mm experimental results ((**a**) $\theta$=10$^\circ$, (**b**) $\theta$=45$^\circ$, and (**c**) $\theta$=80$^\circ$).

**Figure 7:**
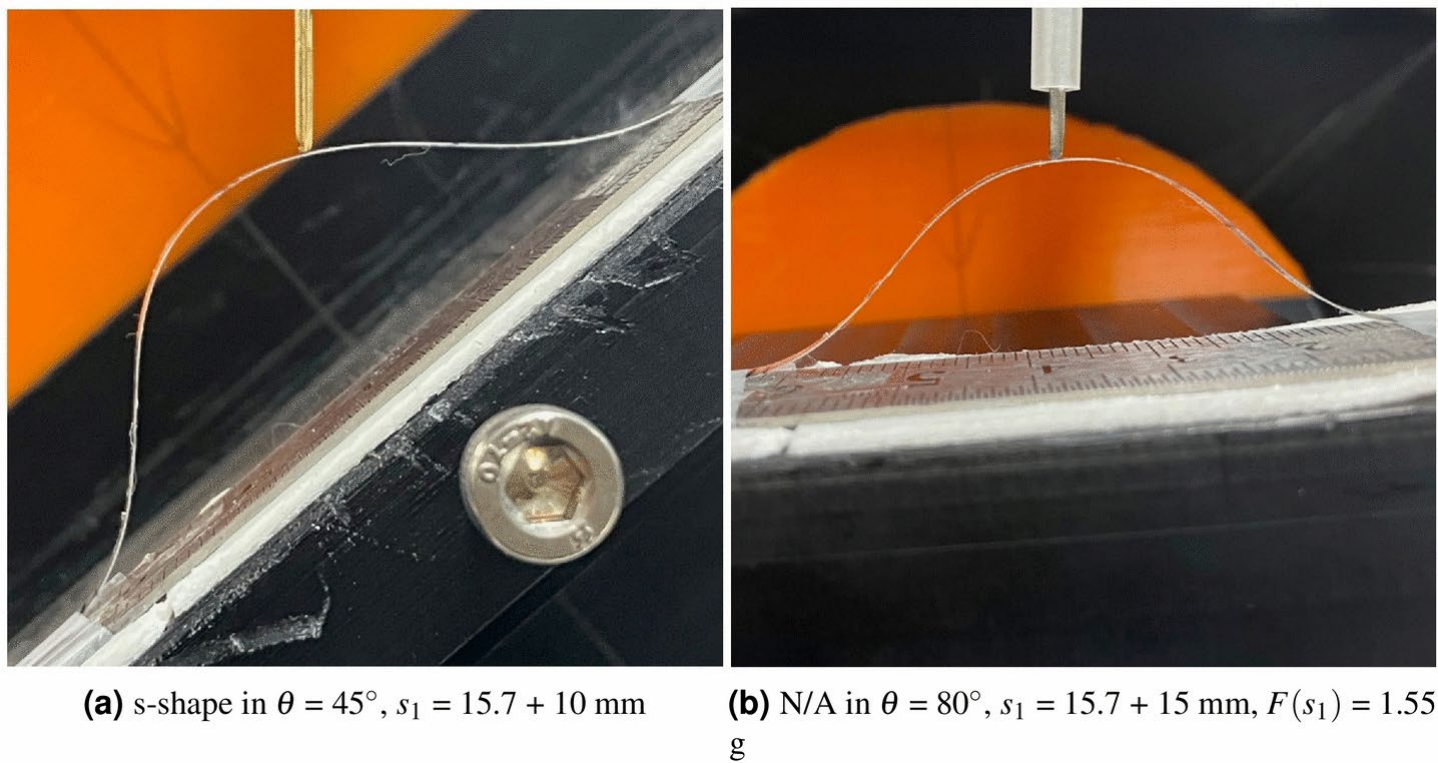
$s_1$ = 15.7 + 10 mm experimental results with (**a**) a s-shaped configuration and (**b**) N/A.

Figure 6a–c present the configuration results when $s_1$ = 15.7 + 5 mm condition. The s-shape configuration in $\theta$ = 45$^\circ$, $s_1$ = 15.7 + 10 mm, $F(s_1)$ = 0.85 g , and N/A in $\theta$ = 80$^\circ$, $s_1$ = 15.7 + 15 mm, $F(s_1)$ = 1.55 g in Table 1 are depicted in Fig. 7a, b, respectively. As can be seen in Fig. 6a–c, all the segment’s configuration is convex shaped, except the 2nd segment in the right picture of Fig. 6c. Note that because of the slipping between the load applying rod and the screen protector, the load applying point A’ in Fig. 2a is not the same as D position in Fig. 2a as can be seen in the right picture of Fig. 6c. In Fig. 7a, s-shape is formulated in the 1st segment. In Fig. 7b, no observable deformation can be seen in the configuration, which results in N/A in Table 1.

Although the problem formulation in Eq. (22) is applicable only under the conditions $s_1$ $\le$ 15.7 + 5 mm, $F(s_1)$ $\le$ *P*, and $\theta$ $\le$ 45$^\circ$, all convex configurations in Table 1 (a total of 12 out of 27 cases) can be modeled using this formulation. For the eight “N/A” cases in Table 1, minimal deformation is observed, as shown in Fig. 7b. Additionally, the three cases exhibiting slight S-shaped configurations can be approximated using the proposed formulation. Consequently, a total of 23 out of 27 cases in Table 1 can be effectively modeled by the problem formulation in Eq. (22). The limitations on the feasible range ( $s_1$ $\le$ 15.7 + 5 mm, $F(s_1)$ $\le$ *P*, and $\theta$ $\le$ 45$^\circ$) arise from the absence of a dedicated classifier to distinguish between convex, slightly S-shaped, and “N/A” cases.

### Preliminary numerical calculation and Ansys simulation results

Based on the experimental results in Table 1, preliminary calculation is performed at $\theta$ = 10$^\circ$ and 45$^\circ$ with *s*1 = 15.7 + 5 mm, $F(s_1)$ = 0.85, 1.55, 2.42 g conditions because the problem formulation in Eq. (22) is feasible in these conditions as explained in previous section. With initial deformation in Fig. 4a, the external force $F(s_1)$ is applied at $\theta$ angle at *s*1 = 5 mm from the end of the 2nd segment.

**Figure 8:**
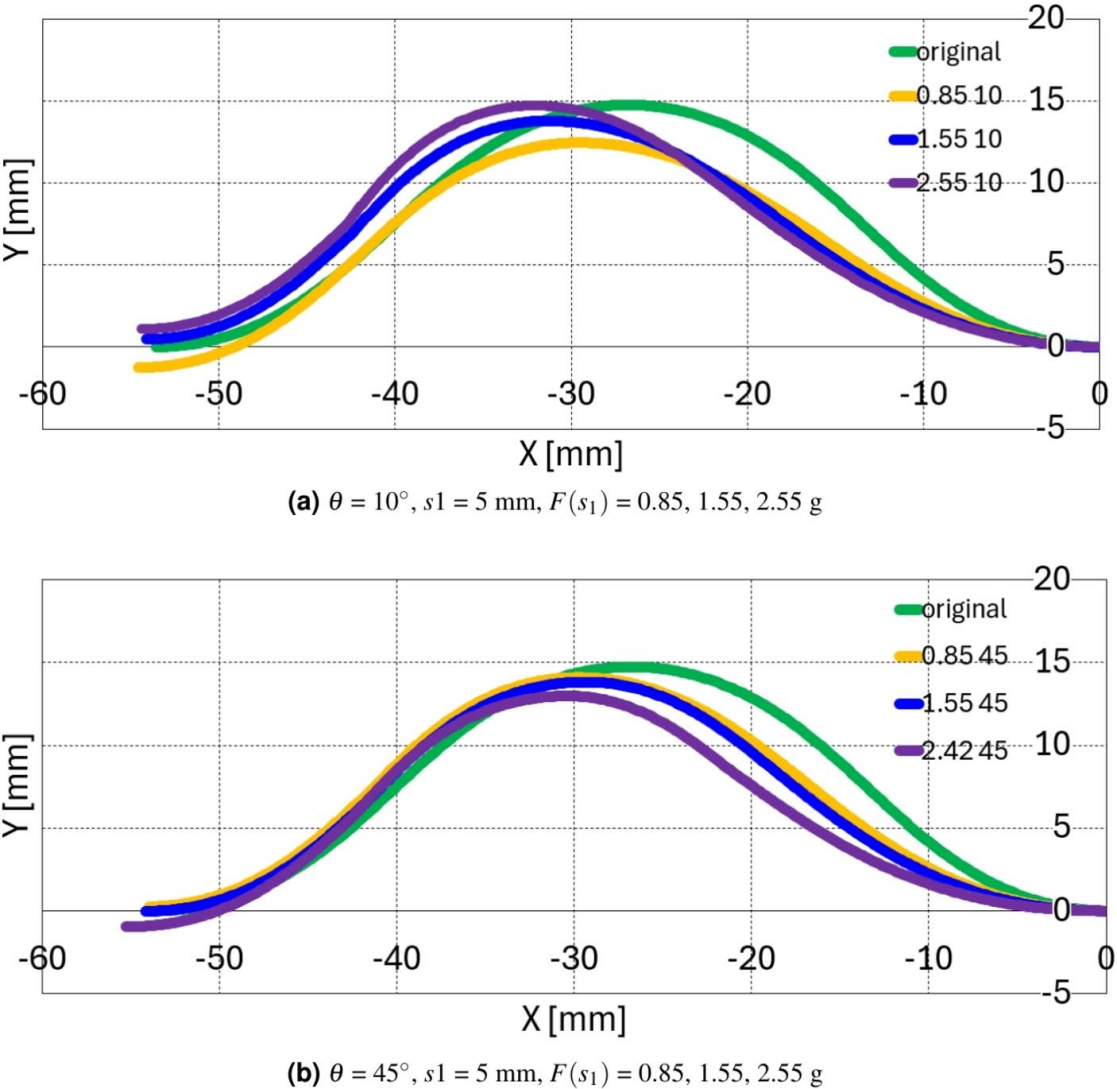
Precalculation configuration result.

In this condition, $\alpha$ $'$, $\beta$, and $\gamma$ are changed from 45 - 30$^\circ$(15$^\circ$) to 45 + 30$^\circ$(75$^\circ$) at 1$^\circ$ increment, and $F_{1x}$ and $F_{1y}$ are changed from 0 to *P* = 2.5 g at *P* / 30 = 0.083 g increment. The four configurations in Fig. 3(a) - (d) are calculated at each ($\alpha '$, $\beta$, $\gamma$, $F_{1x}$, $F_{1y}$) combination with Eq. (10) and connected to render the overall green configuration in Fig. 2a. With the overall configuration, the five constraint errors are calculated by using Eqs. (17)–(21). Out of all the configuration combinations, the closest combinations to the experimental results $\alpha$ $'$, $\beta$, and $\gamma$ in Table 1 are selected and the one with smallest constraint errors out of the selected combinations are chosen, which are summarized in Table 2, 3, Figs. 8a, b.

**Table 2:** Precalculation result (1) - ($\alpha$ $'$, $\beta$, $\gamma$, $F_{1x}$, and $F_{1y}$).

$\theta$	*s*1	$F(s_1)$	result	$\alpha$ $'$	$\beta$	$\gamma$	$F_{1x}$ (mN)	$F_{1y}$ (mN)
10	5	0.85	Precal.	37	35	45	4.1	1.3
			Exp.	38	35	44	–	–
		1.55	Precal.	39	36	50	7.1	2.7
			Exp.	38	34	49	–	–
		2.55	Precal.	39	39	54	11.6	1.6
			Exp.	39	39	54	–	–
45	5	0.85	Precal.	41	40	48	0.28	5.53
			Exp.	41	40	48	–	–
		1.55	Precal.	41	41	48	0.5	6.6
			Exp.	41	41	49	–	–
		2.42	Precal.	38	40	50	0.0	7.4
			Exp.	38	41	51	–	–

**Table 3:** Precalculation result (2)—constraint errors in Eq. (22).

$\theta$	$F(s_1)$	Length error (mm)	*x* error (mm)	*y* error (mm)	Work error (uJ)	Moment error (Nmm)
10	0.85	0.85	1.04	1.26	390.30	0.04
	1.55	0.54	0.24	0.19	20.37	0.06
	2.55	0.39	0.08	0.01	15.82	0.03
45	0.85	0.33	0.21	0.15	16.22	0.03
	1.55	0.35	0.28	0.37	29.17	0.04
	2.42	0.07	0.92	1.44	79.55	0.09

As shown in Table 2, the maximum difference in $\alpha$ $'$, $\beta$, and $\gamma$ values between the precalculations and the experiments is 2 degrees, with an average of 0.5 degrees. The $F_{1x}$ value is much larger than $F_{1y}$ value at $\theta$ = 10$^\circ$, and vice versa at $\theta$ = 45$^\circ$, which is reasonable with respect to the external force applying directions. Table 2 presents the constraint errors in Eqs. (17)–(21), in which average length, x, and y directional errors are 0.42 mm, 0.46 mm, and 0.57 mm, respectively. The average work error (work - deformation energy difference) and moment error are 91.9 $\mu$J and 0.0484 Nmm, respectively. Based on the results of Tables 2 and 3, Eq. (22) to solve the point contact force within *s*1 = 15.7 + 5 mm, $\theta$ = 10, 45$^\circ$, and $F(s_{1})$ = 0.85 g, 1.55 g, and 2.55 (2.42) g seems to be reasonable. The constraint errors presented in Table 3 are attributed to discretization errors, as the parameters $\alpha$ $'$, $\beta$, $\gamma$, $F_{1x}$, and $F_{1y}$ in the precalculation process are confined to a discrete solution space with finite resolution. To minimize verification errors, a high-resolution, fully developed solver must be employed. Incorporating such a solver will enable a more detailed analysis of the modeling error associated with Eq. (22).

Figure 8a, b present the precalculation configuration result of $\theta$ = 10$^\circ$ and 45$^\circ$, respectively. The green, orange, blue, and purple configurations in Fig. 8 presents the original (unforced) configuration, $F(s_{1})$ = 0.85 g, 1.55 g, and 2.55 (2.42) g configurations, respectively. In Fig. 8a, the configuration deforms with decreased $\alpha$ $'$ and $\beta$ values in 1st and 2nd segments and increased $\gamma$ value in 3rd and 4th segment. The overall configuration in Fig. 8a is that the 1st and 2nd segment retreat and the 3rd and 4th segment protrude in the upper right direction as the force increases. In Fig. 8b, the overall configuration retreat to the lower right direction in the 1st and 2nd segment as force increases and presents minor configuration deformation in the 3rd and 4th segment compared with Fig. 8a. All these configuration seems to be consistent with the force applying direction and magnitude.

**Figure 9:**
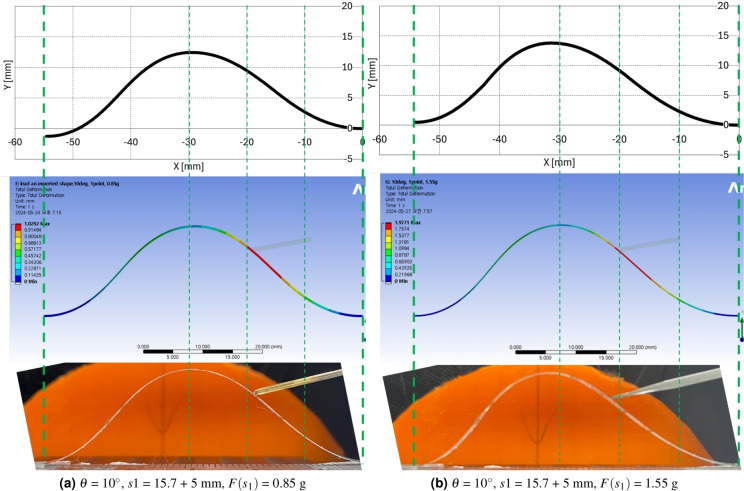
Ansys simulation results with preliminary and experimental calculation results 1 ($\theta$ = 10$^\circ$, *s*1 = 15.7 + 5 mm, $F(s_1)$ = 0.85, 1.55 g).

**Figure 10:**
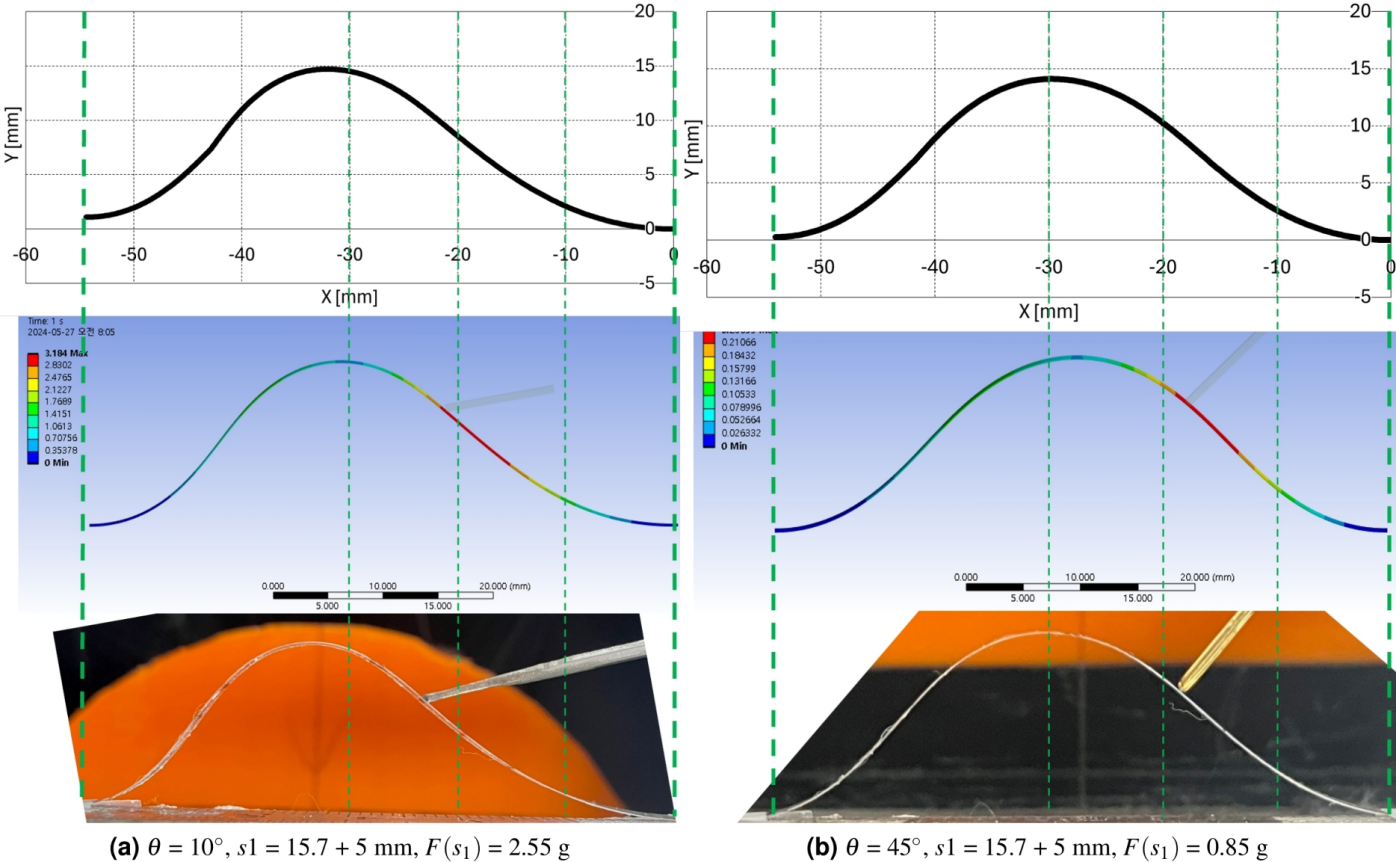
Ansys simulation results with preliminary and experimental calculation results 2 ($\theta$ = 10, 45$^\circ$, *s*1 = 15.7 + 5 mm, $F(s_1)$ = 2.55, 0.85 g).

**Figure 11:**
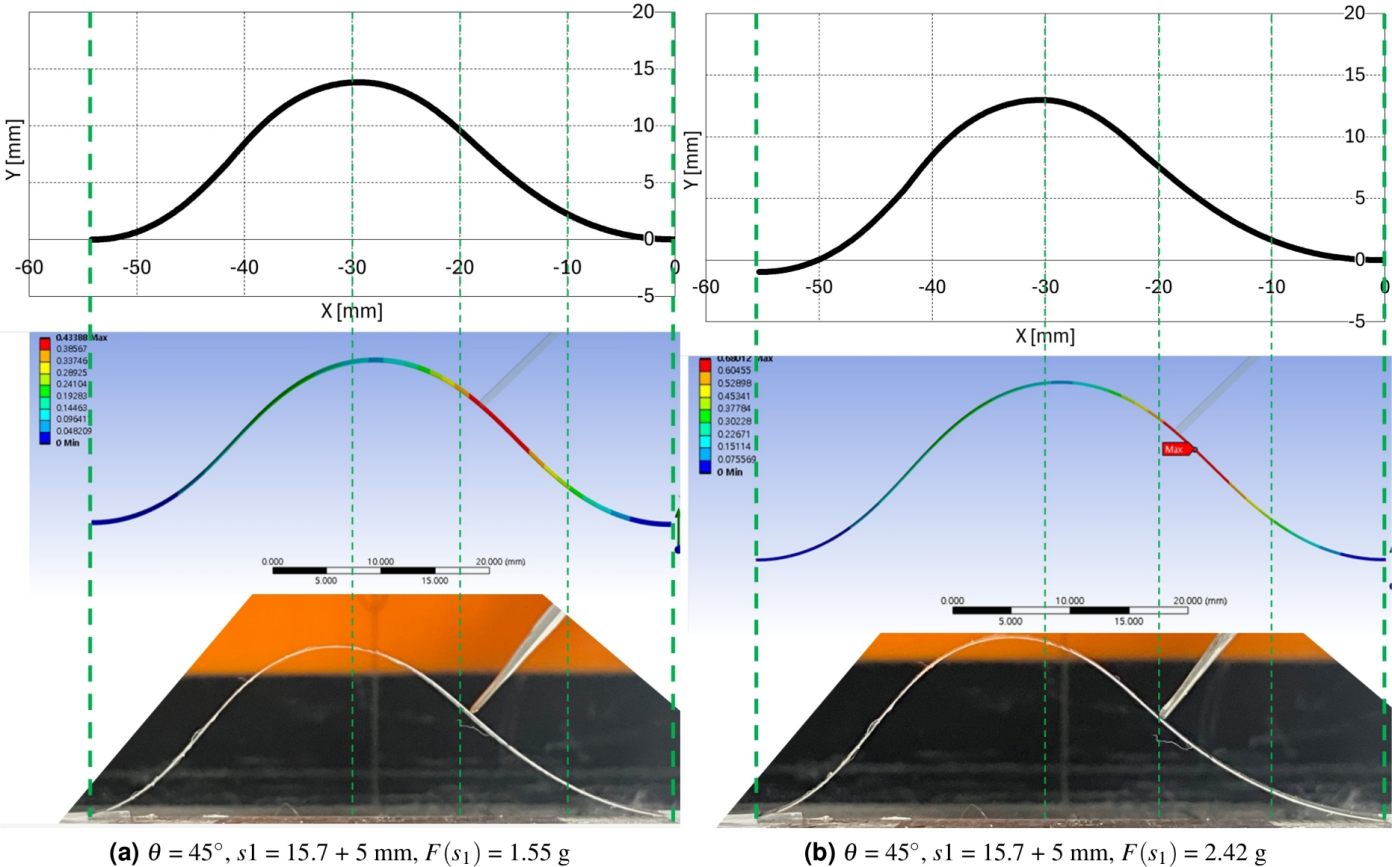
Ansys simulation results with preliminary and experimental calculation results 3 ($\theta$ = 45$^\circ$, *s*1 = 5 mm, $F(s_1)$ = 1.55, 2.42 g).

In order to compare simulation results with the one of preliminary calculation and experiments, numerical simulations using Ansys version 2023 R1 were conducted. Buckling simulations with a screen protector made of Polyethylene were previously performed by Kim et al.^15^, with dimensions of 0.11 mm in thickness, 4.0 mm in width, and 62.95 mm in length. The initial buckling configuration from the previous simulation is exported using the geometry export function in a sub-system of static structural analysis in Ansys. This configuration is then loaded into SpaceClaim, which is one of Ansys’ modeling programs, within another static structural analysis module. Slim rods were designed at each $\theta$ angle of 10$^{\circ }$ and 45$^{\circ }$ to apply force. The total number of nodes and elements for the buckling configuration and each slim rod are 83312 and 28416, respectively. To ensure a dense and consistent mesh at the contact region between the buckling configuration and the slim rod, the face meshing function is employed to generate a quadrilateral mesh on the surface of the buckling configuration. The element size for the buckling configuration is set to 0.00015 through the application of a face sizing function to its entire surface. For the slim rod, an element size of 0.00001 is specified by applying the face sizing function to the edge of the rod at the contact interface with the buckling configuration.

The slim rods are positioned almost precisely at the point s1 = 15.7 + 5 mm, and frictionless contact conditions were applied between the buckling configuration and the rod. Fixed support conditions are applied to both ends of buckling configuration to replicate the real experimental condition, and the forces of 0.83, 1.66, and 2.5 g are respectively applied to the end of slim rod, which does not contact to the buckling configuration. Frictionless support conditions are applied to the surfaces of the slim rod, except for both ends, allowing the rod to move and contact the buckling configuration without any resistance. Total deformation of the screen protector is checked as the simulation result, as shown in Table 4. In Table 4, it is observed that as the force applied by the rod to the buckling configuration increases, the resulting displacement also increases. Additionally, a greater angle at the contact point between the rod and the configuration results in a smaller displacement within the configuration.

**Table 4:** Ansys simulation results.

$\theta$	$s_1$	$F(s_1)$	Total displacement (mm)
10	5	0.85	1.02
		1.55	1.97
		2.42	3.18
45	5	0.85	0.23
		1.55	0.43
		2.42	0.68

The preliminary calculation, Ansys simulation, and experimental results with screen protector are compared in Figs. 9a–11b. Figures 9a, b,  10a, b,  11a, b present the precalculation configuration result of $\theta$ = 10$^\circ$ and 45$^\circ$, respectively. The Ansys results displays the deformation at each point with respect to the original undeformed configuration. The vertical greed dotted lines in Figs. 9a–11b is drawn to compare the three configurations in each subfigures properly. As can be seen in each subfigures, the three results display consistent configurations.

## Conclusion and discussion

In this work, a parametric optimization-based problem formulation is proposed for the online configuration determinaton of a thin film buckling under point contact force. Unlike direct numerical simulations, such as FEM, the proposed method is capable of calculating the configuration online, making it particularly suitable for clinical applications. Furthermore, the incorporation of a regularization strategy to address measurement uncertainties enhances the appeal of this optimization-based approach when compared to traditional analysis methods.

The problem formulation consists of 5 key variables and constraints that must be satisfied for the configuration solution to be feasible with respect to boundary condition, moment continuity, and energy conservation. To verify the proposed problem framework, experiments, Ansys simulations, and precalculations are peformed and compared.

The experiments were conducted with 27 conditions to investigate the actual thin film configuration. The results showed that only 6 conditional results out of 27 satisfied the resultant configuration assumptions of the proposed problem formulation. To meet the assumptions, the force applying position *s*1 must be less than or equals to 5 mm from the beginning of 2nd segment, the force applying angle $\theta$ must be less than or equals to 45$^\circ$, and the force mangitude must be less than or equals to the magnitude of *P*.

After finishing the experiments, precalculations are performed with the feasible 6 condition to compare the results with experimental results. In the precalculation, the 5 variables are changed incrementally, and the overall configuration and 5 constraint errors are calculated. With these calculation results, 6 results are selected with minimm angular difference from the experimental results and constraints errors. The average angular difference of the 6 selected results is 0.5$^\circ$ and average length, x, y directional constraints errors are 0.42 mm, 0.46 mm, 0.57 mm, respectively. Also, the configuration is plotted to verify the overall configuration with force applying direction. The quantitative and qualitative results showed that the proposed problem formulation can be a feasible candidate for the configuration calculation of a thin film buckling under point contact force in *s*1 $\le$ 5 mm, $\theta$ $\le$ 45$^{\circ }$, *F* $\le$ *P*. The Ansys simulation is performed to compare the precalculation and experimental results. The three results are compared visually to validate the proposed problem formulation and they showed consistent results.

For clinical applications, the proposed method can be integrated into the mini basket-type mapping catheter prototype depicted in Fig. 1. This integration, in conjunction with results from previous work^15^, aims to assist clinicians in accurately localizing ECG signals. The tip of the developed mini basket-type mapping catheter, as shown in Fig. 1, incorporates a position/orientation sensor that measures the tip’s spatial orientation relative to the sensor coordinate system. Utilizing the position and orientation of the tip, the relative positions of the electrodes for ECG signal measurement can be determined. This is achieved through a previously established method^15^ in the absence of interactive force and through the proposed method in the presence of point contact force. However, to ensure the full functionality of the mapping catheter, several challenges must be addressed. First, the heart wall must be pre-mapped accurately. Second, the interaction force between the deformed sensor strip and the mapped heart wall must be quantitatively modeled. Lastly, an online configuration determination method for thin-film buckling under distributed forces must be developed to enhance the precision of configuration determination.

There are limitations and issues to be addressed in the future work for applying the proposed methodology, as follows.

1. Although the problem framework in Eq. (22) has been validated, further integration of an appropriate optimization solver, such as Sequential Quadratic Programming (SQP)^35^ or the hybrid projection method^36^, is required to fully achieve online configuration determination of thin film buckling under point contact force. Using the SQP/FSQP (Feasible Sequential Quadratic Programming) algorithm^35,37^, the equality constrained nonlinear optimization problem in Eq. (22) can be solved with relatively high accuracy. However, due to the incremental nature of the feasible constraints, sufficient computation time is required to achieve an accurate solution. Additionally, the FSQP algorithm^37^ includes tunable parameters that can reduce solution accuracy to allow for early termination. The hybrid projection method^36^ alternates between projection and regularization (or vice versa) to minimize the optimization problem, incorporating a regularization term. The accuracy of the solution and the timing of solver termination depend on the magnitude of the regularization parameter and the solver’s termination conditions. To apply the hybrid projection method, the equality-constrained optimization problem in Eq. (22) must be reformulated into an unconstrained optimization problem using either Lagrange multipliers or a penalty function. For the proposed method to function as an online solver, a tradeoff between accuracy and computation time must be carefully considered, and parameter tuning in each solver must be optimized for performance. Furthermore, in the implementation of the solver, the sampling rate of the electrode sensors and actuators in the mapping catheter prototype introduces additional delays, which affect the algorithm’s overall performance. Once the configuration-determining solver is validated with numerical data, a comprehensive performance evaluation should be conducted, incorporating the mapping catheter prototype, to assess the overall system performance.
2. The feasible force application conditions identified in this study (*s*1 $\le$ 5 mm, $\theta$ $\le$ 45$^{\circ }$, and *F* $\le$ *P*) are limited and represent only an approximate condition for applying the problem framework. As solver development progresses, these conditions should be re-examined and extended to ensure a complete set of force application parameters.
3. The initial configuration’s deflection angle of 45$^\circ$, as shown in Fig. 4a, b, was selected to maintain consistency with prior studies, as it represents a median angle between 0$^\circ$ and 90$^\circ$. However, future studies should validate other initial deflection angles to ensure the robustness of the proposed approach across a wider range of initial conditions.
4. The screen protector, made of polyethylene material, is selected as a validation material for the proposed method to maintain consistency with the authors’ previous work^15^. The screen protector has a modulus of elasticity of approximately 19.6 MPa and a Poisson’s ratio of 0.4–0.45. In contrast, the mapping catheter sensor prototype shown in Fig. 1 has a modulus of elasticity of 4.29 GPa and a Poisson’s ratio of 0.34. The dimensions of the screen protector are a thickness of 0.11 mm and a width of 4.0 mm, while the corresponding dimensions for the mapping catheter sensor prototype are a thickness of 0.13 mm and a width of 0.9375 mm, as reported in^15^. Given these differences, it is essential to also validate the proposed method using the mapping catheter sensor prototype to ensure its applicability and reliability in relevant scenarios.

## Data Availability

The datasets used and/or analysed during the current study available from the corresponding author on reasonable request.
